# Experimental Study on the Vegetation Growing Recycled Concrete and Synergistic Effect with Plant Roots

**DOI:** 10.3390/ma12111855

**Published:** 2019-06-07

**Authors:** Fengchi Wang, Chang Sun, Xiangqun Ding, Tianbei Kang, Xiaomei Nie

**Affiliations:** 1 School of Transportation Engineering, Shenyang Jianzhu University, Shenyang 110168, China; 2 School of Civil Engineering, Shenyang Jianzhu University, Shenyang 110168, China; Shiman@stu.sjzu.edu.cn (C.S.); Kangtianbei@stu.sjzu.edu.cn (T.K.); Sxiaomei@stu.sjzu.edu.cn (X.N.); 3 School of Material Science and Engineering, Shenyang Jianzhu University, Shenyang 110168, China; dingxiangqun@sjzu.edu.cn

**Keywords:** vegetation growing recycled concrete, mechanical property, alkali reduction treatment, plant compatibility

## Abstract

Vegetation growing recycled concrete (VGRC) is a relatively new building material that has both biocompatibility and engineering function. The basic performance of VGRC was investigated by experimental analysis, and the hydration products and pore structure of different VGRC mix proportions were studied by X-ray diffraction (XRD), scanning electron microscope (SEM), and industrial computed tomography (CT). The results show that ultrafine slag can reduce Ca(OH)_2_ content in cementing material and has a filling effect on micropores. VGRC has the best performance; the internal pore distribution is uniform when porosity is 20–25%, and the ultrafine slag content is 40%. The compressive strength of VGRC is greatly damaged by the quick-freezing method, while the degree of damage from natural freeze–thaw cycles is relatively small. Soaking in acid solution can effectively reduce the internal pore alkalinity of VGRC. Most plants can grow normally in vegetation concrete, and plant roots can penetrate 6-cm thick concrete blocks after being planted for 60 days. The compressive strength of VGRC decreased after turf planting of 30 days and then increased slowly. The permeability coefficient of VGRC increases with the increase in porosity and aggregate size and decreases after planting and covering. The frost resistance of VGRC is enhanced, and the influence of aggregate size and porosity is small after turf planting.

## 1. Introduction

The traditional construction mode often neglects the co-prosperous relationship between the harmonious development of man and nature, destroying the balance between ecological environment and industrial process. Under the unbalanced development pattern, countries all over the world are facing a series of serious ecological problems, such as urban rainwater, floods, natural water pollution, climate change, and environmental pollution. At the same time, the ground is covered by concrete and other building materials, and the permeability and air permeability of cities are seriously weakened. Vegetation growing recycled concrete (VGRC) is a kind of concrete composed of a sand-free porous recycled concrete skeleton and plant-growing substrate in the pores, which has a certain strength and is suitable for plant growth [1].

Japan first put forward the concept of vegetation growing concrete (VGC) and obtained a number of scientific research results through planned experimental research and exploration [2]. A more mature and perfect construction technology of ecological concrete is put forward. The VGC has been produced with compressive strength of over 10 MPa and a porosity between 22% and 26%, and plants have nicer viewing effect. VGC has been applied to hundreds of practical projects, such as riverbanks, highways, and slopes.

The skeleton of VGC is a kind of porous concrete. Bhutta [3] evaluated the performance of high-performance porous concrete, and the research showed that high-performance porous concrete has excellent working performance. Kevern [4] adopted the prewetting lightweight aggregate (PLWA) method to reduce the cracking and shrinkage of permeable concrete and then evaluated the performance changes of permeable concrete when fine PLWA completely replaced sand in permeable concrete. Kyung [5] replaced ordinary Portland cement with cement containing α-hemihydrate calcium sulfate and developed a low-alkalinity neutral bonding material, which can effectively reduce the alkalinity of vegetation concrete pores. Mohammed [6] studied the effects of nanosilica (NS) and fly ash (FA) on the basic properties of pervious concrete. Oh RO [7] found that replacing some cementitious components with alkaline activators and natural jute fibers could improve the compressive strength, porosity, water purity, and corrosion resistance of VGC. Ghashghaei [8] found that the aggregate size and water cement ratio are important factors affecting the permeability of porous concrete, and established a numerical model for predicting the permeability coefficient of porous concrete. Huang [9] believed that the method of sealing porous concrete by vacuum packaging can measure the porosity of pervious concrete more accurately. Lian and Jang [10,11] thought that the compressive strength of porous concrete has a great correlation with the porosity and fits the regression equation mathematical model through theoretical deduction, which could predict the compressive strength of concrete under the conditions of set porosity. Sumanasooriya [1,12] found that adding admixtures not only improves the porosity of vegetation concrete but also reduces its water permeability and compressive strength. Codina [13] developed a low alkalinity cement suitable for VGC, which can adjust the concrete internal alkalinity to a suitable condition for vegetation growth. Xu [14] replaced part of the cement with fly ash to reduce the pore alkalinity of concrete. It was found that adding low calcium fly ash to ordinary Portland cement could significantly reduce the hydration heat and the number of Ca(OH)_2_ crystals after hydration. Räsänen [15] proposed a simple pH value determining method, which can quickly and effectively measure the internal alkalinity of VGC. Wong [16] found that grass roots can penetrate concrete pores and extend into underlying soil after vegetation planting tests, which had no expansion damage to the VGC, and could improve the integrity of vegetation concrete slope protection.

The use of recycled aggregates (RAs) is essential for realizing a sustainable construction industry. Studies of RAs applied to concrete are significant in preserving the environment and the effective use of resources worldwide [17]. In recent years, there have been a great number of studies on the recycling of aggregates in the structural concrete manufacture. The old mortar particles adhere to RAs with relatively high porosity and low strength, weakening the cementation of material, which leads to the inferior properties of RAs compared with NAs [18,19]. The application of RAs can increase the permeability of vegetation concrete. Yap [20] and Rahman [21] studied the mix ratio of recycled building materials and the constructed permeable pavement system, focusing on the study of water permeability and mechanical properties. Gelong [22] applied Scattering-filling coarse aggregate (SFCA) process to prepare the recycled aggregate concrete with promoted performance. The results indicated that SFCA process could improve the compressive strength and elastic modulus and reduce the drying shrinkage and chloride penetration. Therefore, it is feasible to use RAs instead of NAs in pervious concrete, which can meet the requirements of mechanical properties, ionic penetration resistance, and frost resistance.

Based on the existing research results, in this paper, recycled aggregate was used instead of coarse aggregate in the VGC, and ultrafine slag was used to reduce the amount of cement in the cementitious materials. This can consume urban construction waste, reduce the pore alkalinity, and realize the permeable greening function of VGRC. The objective of this study is to investigate the mechanical properties and frost resistance of the vegetation growing recycled concrete and synergistic effect with plant roots. Firstly, by studying the influence of the cementitious material components, aggregate properties, water cement ratio, porosity, and other factors on the basic properties of concrete, such as compressive strength, tensile properties, and frost resistance, the optimum proportion of vegetation recycled concrete products is determined (Section 3). Meanwhile, the CT scanning technology was carried out to reflect the internal porosity of VGRC (Section 4). Then, the compatibility of vegetation concrete with lawn plants was evaluated by planting tests after alkali reduction treatment and planting base material filling, in order to provided technical support and theoretical reference for the engineering applications of vegetation recycled concrete (Section 5 and Section 6).

## 2. Materials and Methodology

### 2.1. Specimen Making

**Figure 1:**
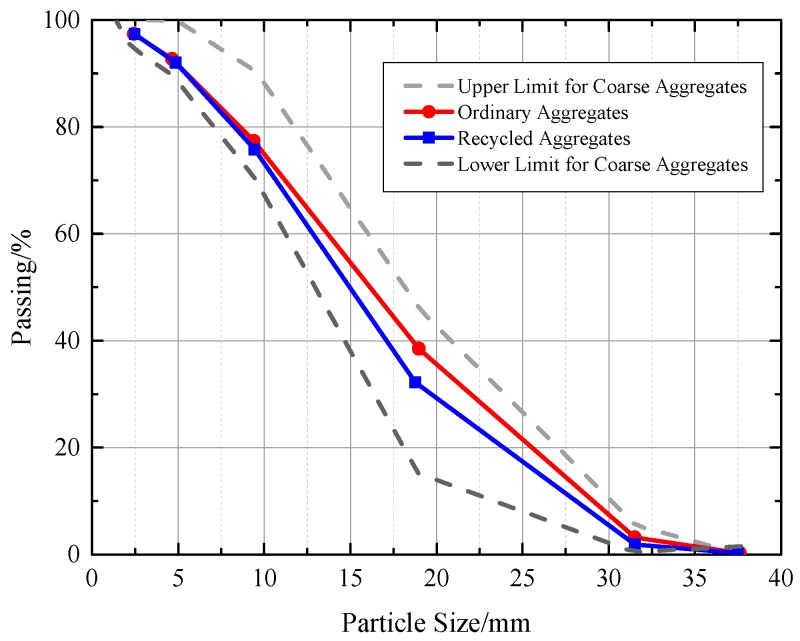
Coarse Aggregates Gradating Curves.

The raw materials used in the experiment mainly include porous pervious concrete materials, which constitute the framework structure of vegetation recycled concrete, chemical reagents used in alkali reduction treatment, planting substrates and lawn seeds selected in the planting experiment. The concrete skeleton material was P.O 42.5 ordinary Portland cement. Recycled aggregates from waste concrete beams with strength of C40 were produced after crushing and screening by a jaw crusher. The ordinary aggregates are natural gravels. The properties of the coarse aggregates were tested according to GB/T 50081 and the performance indicators are shown in Table 1. The grain size distribution curves of the coarse aggregates are shown in Figure 1. Ultrafine slag with 520 m^2^/g specific surface area was obtained from crushing ore powder. The chemical composition of slag is listed in Table 2. In addition, a liquid poly carboxylic acid water reducer was used. The chemical reagents used were oxalic acid and ferrous sulfate. Planting substrates used were coconut bran, peat soil, polyacrylamide water-retaining agent, powdery agricultural potassium salt water-retaining agent, and organic fertilizer.

**Figure 2:**
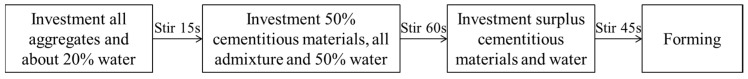
Operation Process of Slurry Wrapping Method.

The mix design method of VGRC was referred to pervious concrete. The amount of aggregate and cementitious material per unit volume was calculated by density after removing the pore volume per unit volume. The water-cement ratio was determined according to the test requirements, and the dosage of mixing water and cement was further determined. The mixing technology was the slurry wrapping method, using a forced single horizontal shaft concrete mixer. The operation method is shown in Figure 2. The mixture proportions of specimens are listed in Table 3.

**Figure 3:**
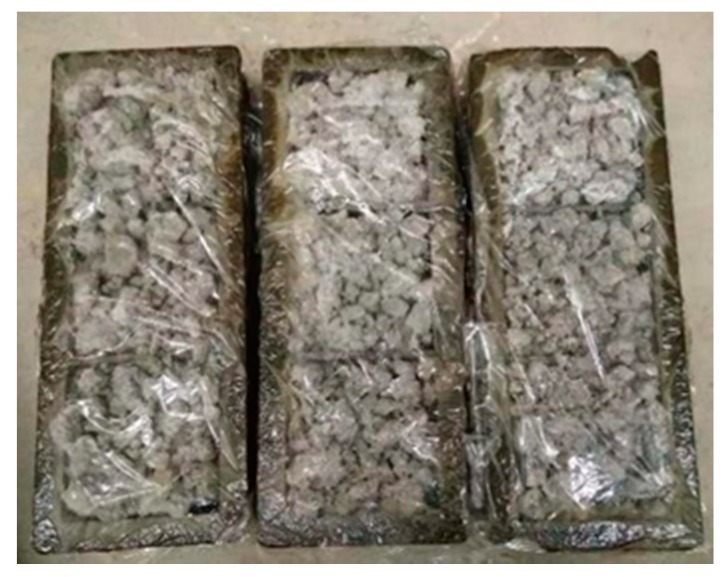
Maintenance Methods of Specimens.

Vibration and insertion-tamping were used in the forming process. Three layers of concrete were loaded into the mold, and each layer was tamped densely. After filling, the concrete was placed in a shaking table to vibrate for 8 to 10 s. Membrane moisturizing was adopted in the maintenance way (see Figure 3); that was wrapping plastic film over the exposed surface of the top of the mold after the formwork was completed and keeping the specimen in a standard maintenance room at constant temperature and humidity until the required age.

### 2.2. Test Method

The test method of the compressive strength refers to GB/T50081-2002 Standard for Test Method of Mechanical Properties of Ordinary Concrete. The cubic compressive specimens with a side length of 100 mm were cured to 28 days age under standard conditions and loaded by a MYL-2000D pressure-testing machine (JianYi, Wuxi, China) with a loading speed of 0.5–1.5 MPa/s.

The test method of splitting tensile strength refers to GB50081-2002 Standard for Test Method of Mechanical Properties of Ordinary Concrete. The loading speed was 0.02–0.05 MPa/s. The splitting tensile strength of the cylinder was calculated by Formula (1).

(1) $$f_{\alpha }=\frac{2F}{\pi \cdot d\cdot l}=0.637\frac{F}{A}$$

In the formula, *f_α_* is the splitting tensile strength of a cylinder (MPa); *F* is the failure load of the specimens (N); *d* is the diameter of the specimens on the splitting surface (mm); *l* is the height of the specimens (mm); and *A* is the splitting surface area of the specimens (mm^2^).

**Figure 4:**
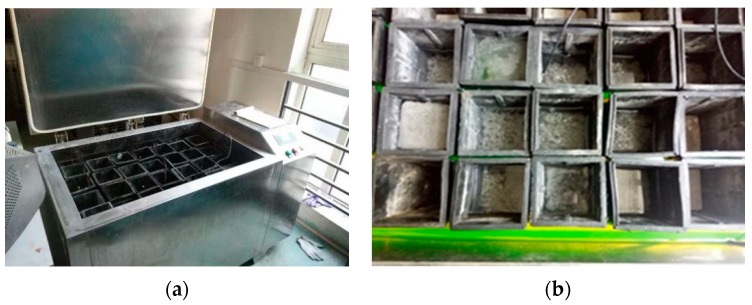
Freezing and Thawing Test Device. (**a**) Freeze-thaw cycle machine; (**b**) Arrangement of freeze-thaw cycle specimens.

The test method of the freeze-thaw cycle refers to GB/T50082-2009 Standard for Test Methods of Long-term Performance and Durability of Ordinary Concrete. The cycle index was 50 times using a KDR-V freeze-thaw cycle machine (see Figure 4).

The porosity was measured by the drainage method. The cubic specimen with a side length of 100 mm was immersed in water for 30 min. After fully absorbing the water, the specimen was taken out to drain the surface water and placed in a single open container of equal volume. Using a measuring cylinder to hold 1000 mL water, it was slowly injected into the container until it was full. The volume of the injected water is the pore volume of specimen.

**Figure 5:**
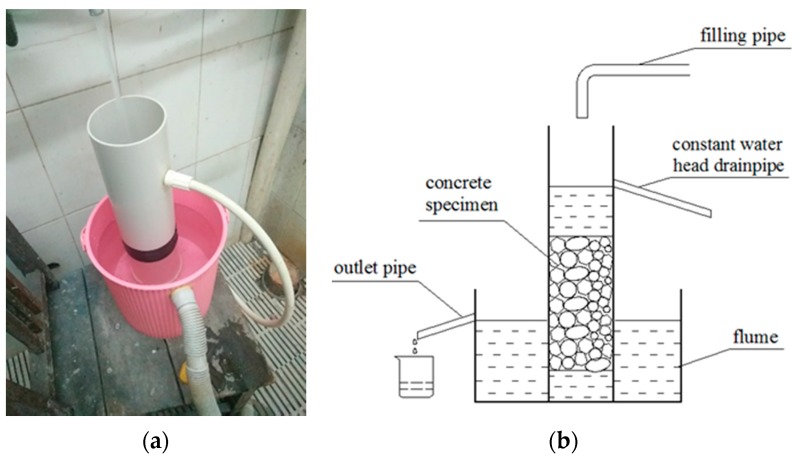
Permeability Coefficient Measuring Device and Schematic Diagram. (**a**) Permeability coefficient measuring device; (**b**) Schematic diagram.

The test method of the permeability refers to CJJ/T 135-2009 Technical Specification for Pervious Cement Concrete Pavement. A homemade improved concrete permeability coefficient measuring device was adopted (see Figure 5). Under the condition of unchanged water pressure, the water quality of passing through the VGRC in a certain time was measured, and the permeability coefficient was calculated according to Formula (2).

(2) $$K_{T}=\left(Q\times T\right)/\left(H\times A\times t\right)$$

In the formula, *K_T_* is the permeability coefficient at *T* °C water temperature (mm/s); *A* is the osmotic compressive area of the concrete (mm^2^); *Q* is the amount of water seeping out in *t* seconds (mm^3^); *H* is the water level difference (mm); and *t* is the testing time(s).

**Figure 6:**
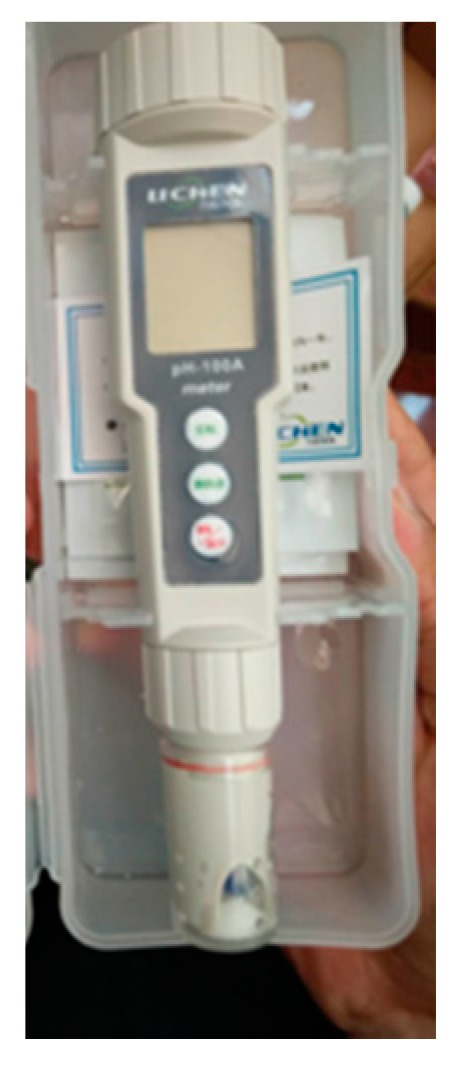
Pen Acidometer.

The pore pH value of the VGRC was determined by the alkalinity release method. The cubic concrete blocks with a side length of 10 cm were put into the container with 4000 mL of pure water. The water was changed after soaking 4 h until the pH remained unchanged. The water pH value is the pore pH value, which was measured by a pen acidometer (see Figure 6). The pH value determination method of plant substrates refers to NY/T1377-2007 Determination of pH in Soil.

**Figure 7:**
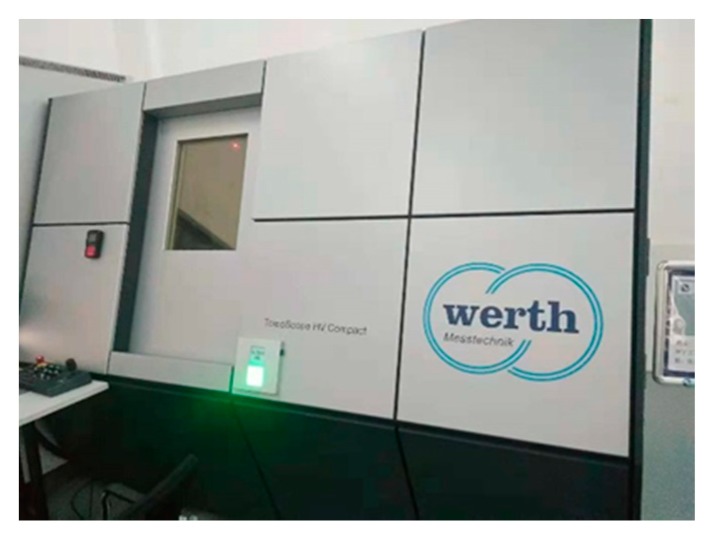
X-ray Tomography Coordinate Detector.

The pore structure was analyzed by a TomoScope HV Compact X-ray tomography coordinate detector (Hannover, Germany, see Figure 7). The pore volume, pore size, pore morphology, and other related data can be obtained through VG Studio MAX3.0 software.

## 3. Study on Mechanical Properties of Vegetation Growing Recycled Concrete (VGRC)

### 3.1. Mechanical Properties of Ultrafine Slag Cement Mortar

**Figure 8:**
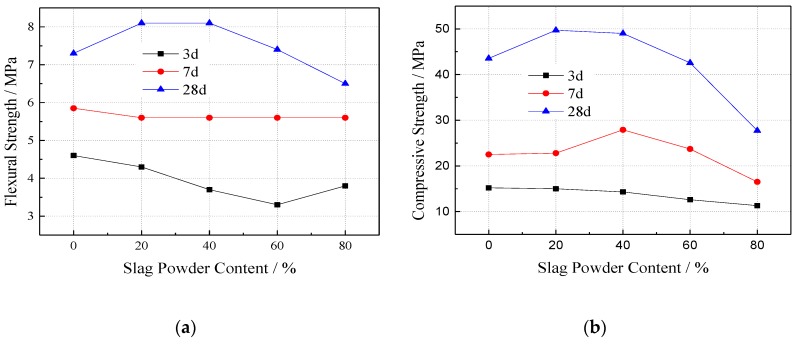
Experimental Results of Cement Mortar Strength. (**a**) Effect of slag content on flexural strength of cement mortar; (**b**) Effect of slag content on compressive strength of cement mortar.

Ultrafine slag is a kind of high-fineness and high-activity powder obtained from water-quenched blast furnace slag through the drying and grinding processes. It is a high-quality and recognized mineral admixture for concrete. Adding it as a cementitious component to VGRC can reduce cement consumption and improve the pore alkali environment. The mortar strip test was carried out by replacing cement components with the ultrafine mineral powder according to mass ratios of 0, 20%, 40%, 60%, and 80%. The flexural and compressive strength of the mortar at 3, 7, and 28 days were tested; results are shown in Figure 8. The curves show that the flexural and compressive strength of the cement mortar with an ultrafine slag content of more than 60% is lower than that of the blank group at 28 days, which indicates that the mechanical properties of the ultrafine slag is worse than that of ordinary Portland cement and that the dosage of the ultrafine slag should not be more than 60%. The strength of the samples mixed with ultrafine slag is lower than that of the control sample at 3 days and higher or close to the control sample at 7 days, which indicates that the hydration rate of the cementitious system decreases after adding ultrafine slag.

**Figure 9:**
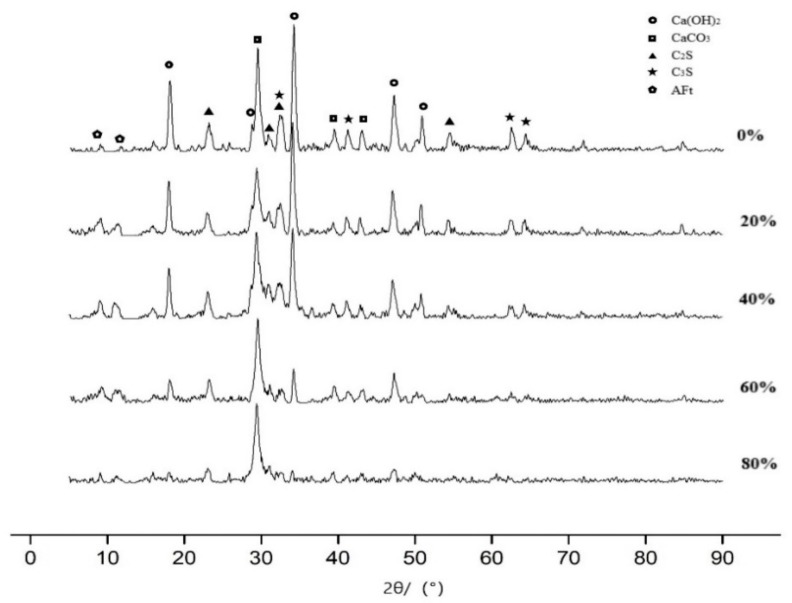
XRD Analysis of 28d Sample of Pure Slurry.

**Figure 10:**
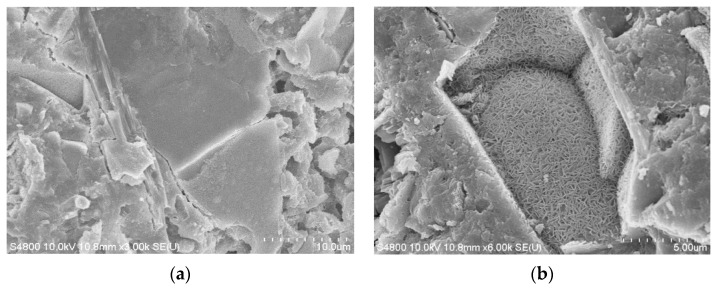
Surface Morphology of Vegetated Concrete. (**a**) Morphology of 28d with 20% of the sample; (**b**) Morphology of 28d with 60% of the sample.

X-ray diffraction (XRD) analysis (SHIMADZU, Kyoto, Japan) of cement pastes with different ultrafine slag content (see Figure 9) shows that the hydration products of cement are mainly Ca(OH)_2_, CaCO_3_, C_2_S, C_3_S, and AFt. The characteristic peak strength of Ca(OH)_2_ decreases obviously with the increase of the ultrafine slag content, which confirms the feasibility of reducing the alkalinity of the ultrafine slag. The characteristic peak strengths of C_2_S and C_3_S in the system decrease with the increase of the content of the ultrafine slag, which indicates that the potential of the system to continue hydration also decreases with the increase of the content of ultrafine slag. It can be seen by electron microscope scanning (see Figure 10) that a large number of hexagonal flake Ca(OH)_2_ crystals and stripy AFt crystal regions exist in the paste samples with 20% ultrafine slag. However, there is no crystal region in the sample with 60% content, although irregular ultrafine slag particles without hydration can be observed. This shows that the addition of ultrafine slag can consume Ca(OH)_2_ crystals in the system through a secondary hydration reaction and play a filling role to make the structure of the cement paste compact.

### 3.2. Compressive Strength

**Figure 11:**
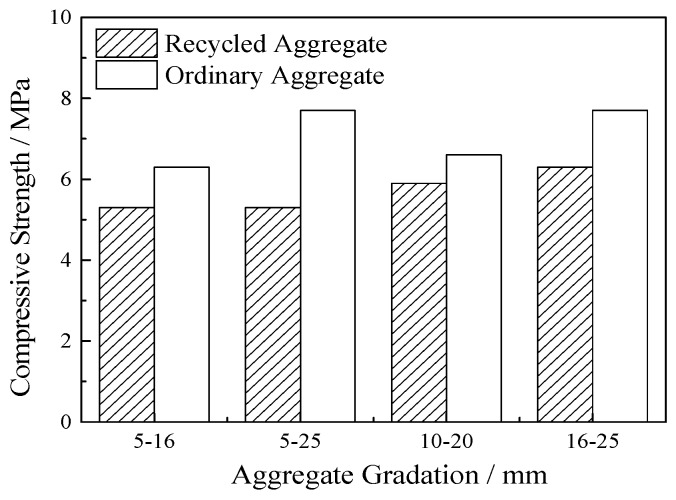
The Influence of Aggregate on Compressive Strength.

Under the conditions of 25% porosity, 60% ultrafine slag, and a 0.25 water-cement ratio, the VGRC mixture ratio was set with the aggregate size as variable. Experiments were carried out with ordinary aggregate and recycled aggregate. The results (see Figure 11) show that the strength increases with the increase of particle size and the compressive strength of the ordinary aggregate is obviously better than that of the recycled aggregate. The recycled aggregate surface is rough, the shape has more edges and corners than limestone gravel, the proportion of needle-like aggregate is higher, and the contact points are less when stacking [23]. Recycled aggregate has undergone a long period of mechanical wear and chemical erosion, and the mechanical damage caused by the crushing process causes more microcracks in the aggregate, which leads to its poor performance as a concrete aggregate when compared with natural aggregate [24]. Furthermore, the smaller sized aggregate makes it easier to produce a stress concentration in the process of skeleton structure under pressure load, which leads to it being more vulnerable to damage.

**Figure 12:**
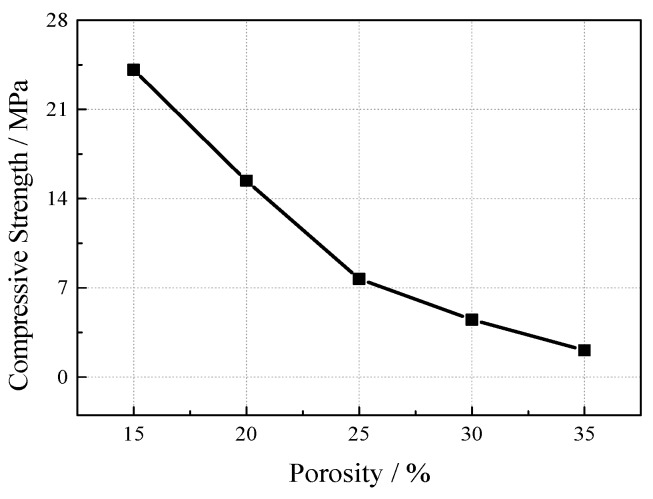
The Influence of Porosity on Compressive Strength.

Five groups of specimens with porosities of 15%, 20%, 25%, 30%, and 35% were set up under the conditions of 40% ultrafine slag and a 0.25 water-cement ratio. The test results (see Figure 12) show that the compressive strength of VGRC decreases with the increase of porosity, and the reduction range is obvious and large. The properties of VGRC are similar to ordinary concrete. The macroscopic strength of the concrete is determined by the number and structure of internal pores. With the decrease of porosity, the voids between the aggregates are gradually filled by cement paste, which reduces the internal structural defects and makes the stress distribution more uniform. In addition, as the proportion of the pore area on the cross section decreases, the effective area under actual stress increases. According to the criteria of VGRC slope protection proposed by Japan, the compressive strength of VGRC for engineering should be kept above 10 MPa, so it is appropriate to control the porosity to between 20% and 25%.

**Figure 13:**
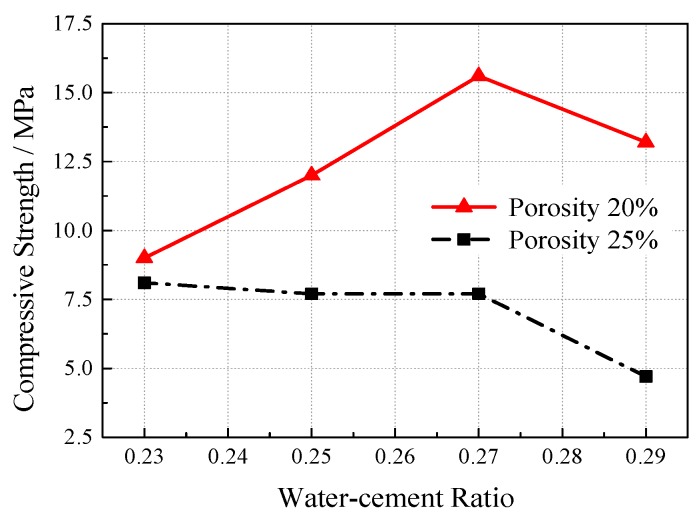
The Influence of Water–cement Ratio on Compressive Strength.

Four groups of specimens with water-cement ratios of 0.23, 0.25, 0.27, and 0.29 were set up under the conditions of 40% ultrafine slag and 20% and 25% porosity. The cement slurry can evenly encapsulate the aggregate without flowing and the cohesion by adjusting water reducing agent dosage to control slurry fluidity was at 170 mm to 220 mm. The results of the compressive strength test (see Figure 13) show that the compressive strength of VGRC is greatly influenced by porosity, which is much higher than that of the water cement ratio. When the porosity is 25%, the cement slurry layer wrapped on the aggregate surface is thinner, and the joint failure leads to the structure failure, which results in the slurry being unable to exert its actual strength. When the porosity is 20%, the effect of the porosity on the compressive strength is small and the number of bonding points between the aggregates increases. The strength provided by the cementitious materials can be fully utilized, which can better reflect the influence of the water–cement ratio on the compressive strength.

**Figure 14:**
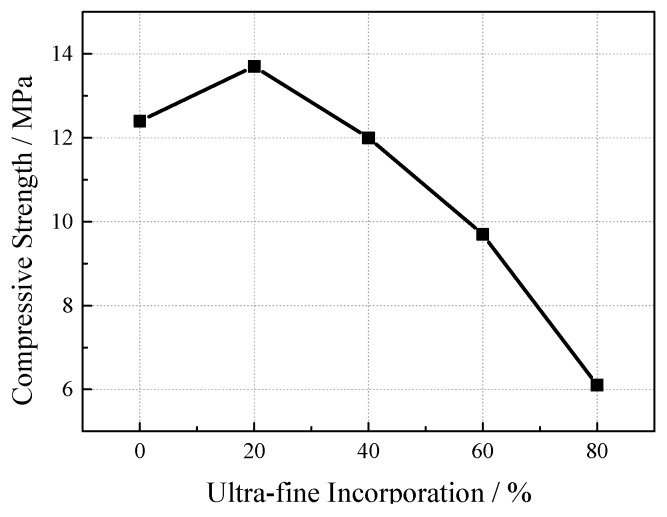
The Influence of Ultrafine Slag Incorporation on Compressive Strength.

Specimens with different ultrafine slag contents were set up under the conditions of 25% porosity and a 0.25 water-cement ratio (see Figure 14). It can be seen from the measured results that with the increase of ultrafine slag, the compressive strength of the concrete first increases and then decreases. On the one hand, the introduction of ultrafine slag can promote the secondary hydration of the cement and make the spatial distribution of hydration products more uniform and compact. Ultrafine slag has unique ultrafine powder characteristics and a micro aggregate effect [25]. It can refine the pore size of slurry, reduce the number of connected holes, improve the compactness, and improve the structure of the interface transition layer. On the other hand, ultrafine slag reacts with calcium hydroxide produced in mixed cementitious system during hydration. The reaction forms a hydrated calcium silicate compound, which can improve the strength of cementitious material, reduce the bond damage between cementitious material and coarse aggregate, and thus improve the VGRC strength. However, when the content of the ultrafine slag is too high, the proportion of cement clinker in the cementitious materials decreases and the hydration rate of cement decreases.

### 3.3. Splitting Tensile Strength

**Figure 15:**
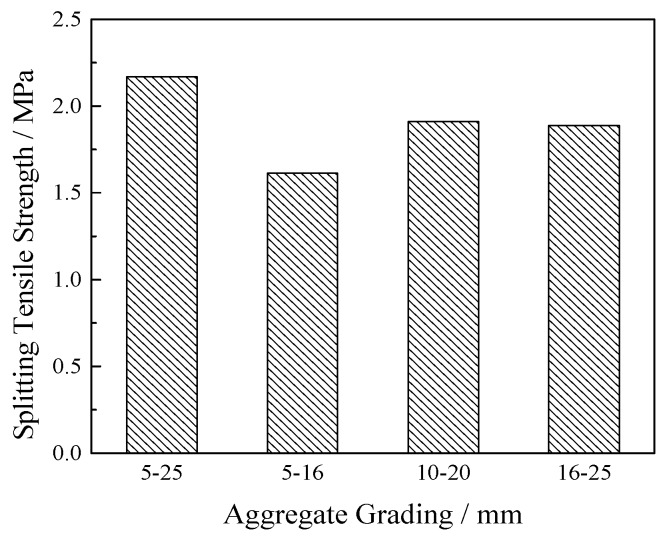
Effect of Aggregate-level Pairing Splitting Tensile Strength.

Under the conditions of 60% ultrafine slag, 20% porosity, and a 0.25 water-cement ratio, VGRC specimens with different aggregate sizes were set up for the splitting tensile test. The data in the figure (see Figure 15) show that the concrete with aggregate gradation of 5–25 mm has the highest splitting tensile strength and the concrete with aggregate gradation of 5–16 mm has the lowest splitting tensile strength. Under the same porosity, the larger the aggregate size is, the greater the tensile strength is. This is because the larger the particle size, the better the recycled aggregate quality is, there are less internal defects and the harder it is to be pulled off.

**Figure 16:**
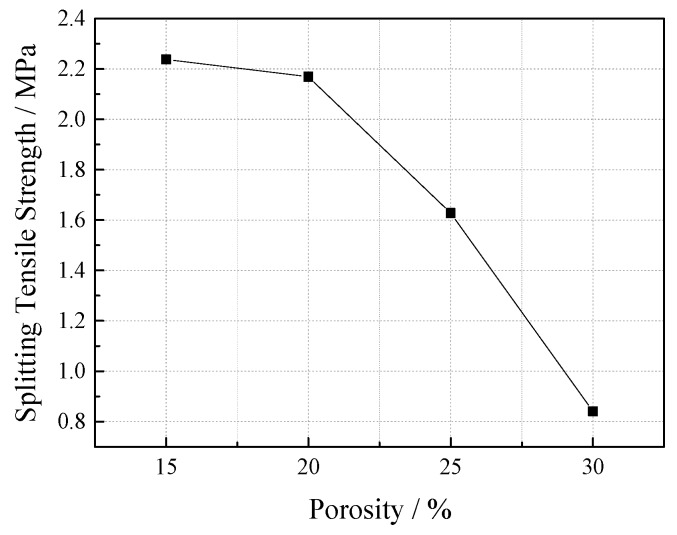
Effect of Porosity on Splitting Tensile Strength.

Four groups of specimens with a porosity of 15%, 20%, 25%, and 30% were set up under the conditions of 60% ultrafine slag and a 0.25 water-cement ratio. The results (see Figure 16) show that the splitting tensile strength of VGRC decreases with the increase of porosity, and the larger porosity is, the greater the splitting tensile strength is reduced.

### 3.4. Frost Resistance Performance

VGRC specimens with a size of 100 mm × 100 mm × 100 mm, an aggregate size of 5–25 mm and an ultrafine slag content of 60% were fabricated to analyze the loss of mass and strength after 25 or 50 freeze-thaw cycles under different water–cement ratios and porosity conditions. The experimental proportions and results are shown in Table 4.

**Figure 17:**
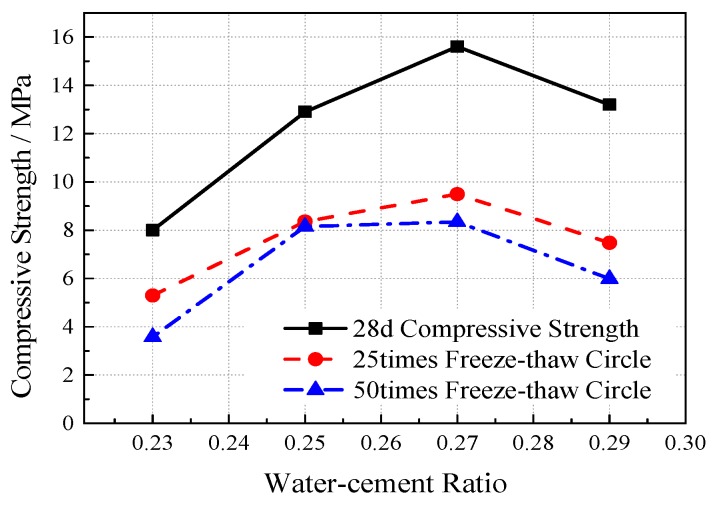
The Influence of Water-cement Ratio on Compressive Strength after Freeze-thaw.

**Figure 18:**
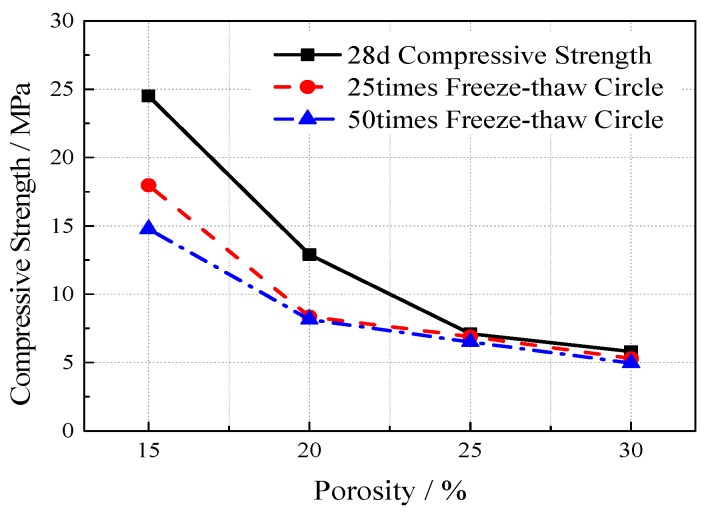
The Influence of Porosity on Compressive Strength after Freeze-thaw.

According to the mass loss rate, when the freeze-thaw cycles occur 25 times, the concrete has almost no aggregate shedding and the mass loss rate of the different mix ratio specimens is less than 3%. When the freeze-thaw cycles are at 50 times, a small amount of the aggregate falls off and the mass loss rate is less than 6%, while the influence of different mixing ratios on the mass loss is small. From the strength changes after freeze-thaw (see Figure 17 and Figure 18), the compressive strength of concrete after freeze-thaw increases first and then decreases with the increase of the water-cement ratio. The compressive strength of concrete generally decreases with the increase of freeze-thaw cycles. After 25 freeze-thaw cycles, the strength decreases by 2.7–6.1 MPa when compared with the initial strength. After 50 freeze-thaw cycles, the strength decreases by 4.4–7.2 MPa. The compressive strength of concrete after freeze-thaw decreases with the increase of porosity and freeze-thaw times and the impact of freeze-thaw times is relatively small. When the porosity reaches 25%, the weakening effect of freeze-thaw on the strength obviously decreases, and the compressive strength after different freeze–thaw cycles is basically the same as the initial strength.

**Figure 19:**
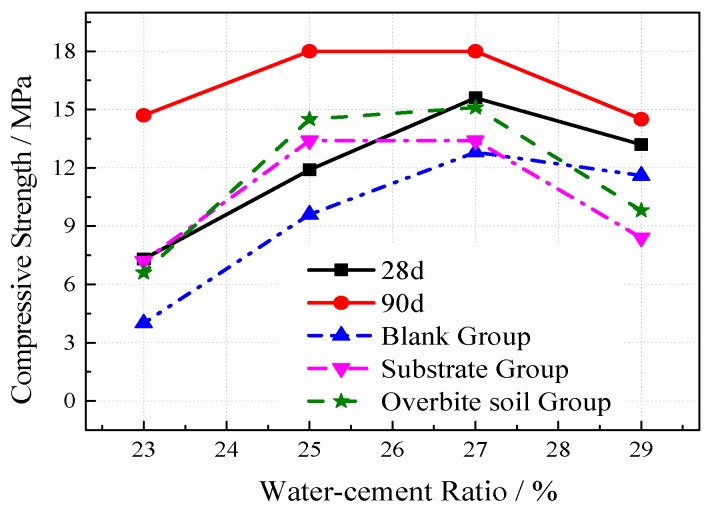
The Influence of Water-cement Ratio on Vegetation Growing Recycled Concrete (VGRC) Frost Resistance.

**Figure 20:**
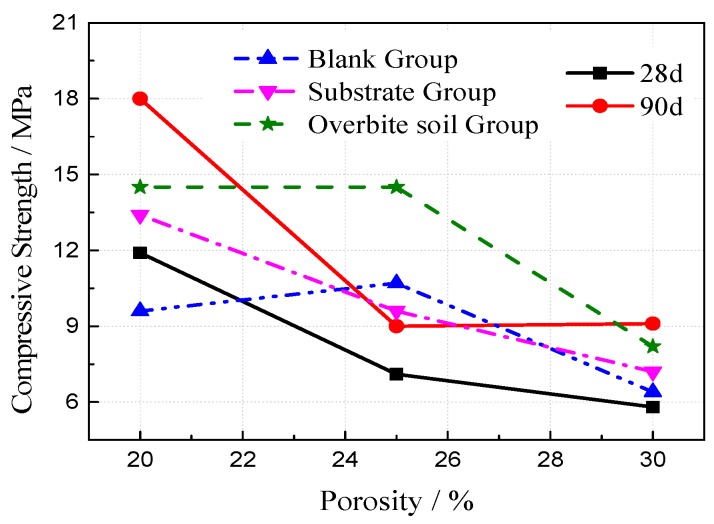
The Influence of Porosity on Vegetation Growing Recycled Concrete (VGRC) Frost Resistance.

The outdoor slow freezing test was set up to study the destructive effect of freeze–thaw on the VGRC in natural conditions. The designed specimen size was 100 mm × 100 mm × 100 mm, the aggregate size was 5–25 mm, the ultrafine slag content was 60%, the porosities were 20%, 25%, and 30%, and the water-cement ratios were 0.23, 0.25, 0.27, and 0.29. The strength change situation after freeze-thaw in the reference group, intact group (i.e., blank group), perfusion pore substrate group (i.e., substrate group), and filling substrate and burying in soil group (i.e., overbite soil group) were investigated. The experimental results (see Figure 19 and Figure 20) show that the effects of the water-cement ratio and porosity on frost resistance of the VGRC in a natural environment are similar to those obtained by the quick freezing method. The loss of compressive strength caused by the slow freezing method is relatively small. The VGRC still has high hydration activity after curing for 28 days, and its strength is still in a growing process. An outdoor environment provides an anhydrous freeze-thaw test, making the concrete failure mode different from that of the quick freezing method. The outdoor freeze-thaw test block is not subjected to the frost heave pressure generated by the freezing process of pore water, so the strength loss is small.

## 4. Pore Analysis and 3D Reconstruction

### 4.1. Pore Spatial Morphology and Parameter

**Figure 21:**
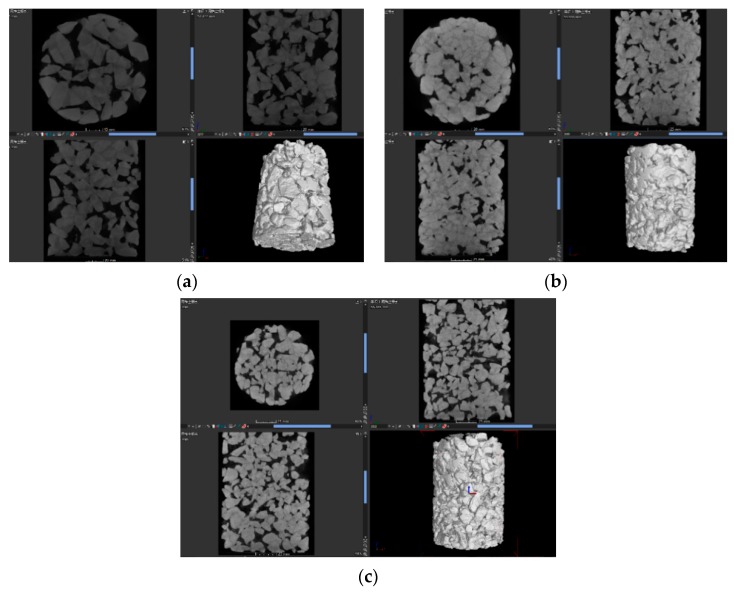
3D Image of Vegetation Growing Recycled Concrete (VGRC) Specimen. (**a**) CT-1; (**b**) CT-2; (**c**) CT-3.

CT scanning technology can accurately obtain the distribution of aggregate and pore structure in concrete without affecting the specimens’ morphology and internal structure [26]. Three mix ratios (see Table 5) were set up for the cylindrical VGRC specimens with a diameter of 10 cm on the bottom and a height of 15 cm. The internal aperture shape was analyzed by an X-ray tomography coordinate measuring machine. The instrument parameters are shown in Table 6. The volume model was generated from the CT data scanned, and the tangential images of the front, side, and elevations of the specimen can be obtained by importing VG Studio software (see Figure 21).

**Figure 22:**
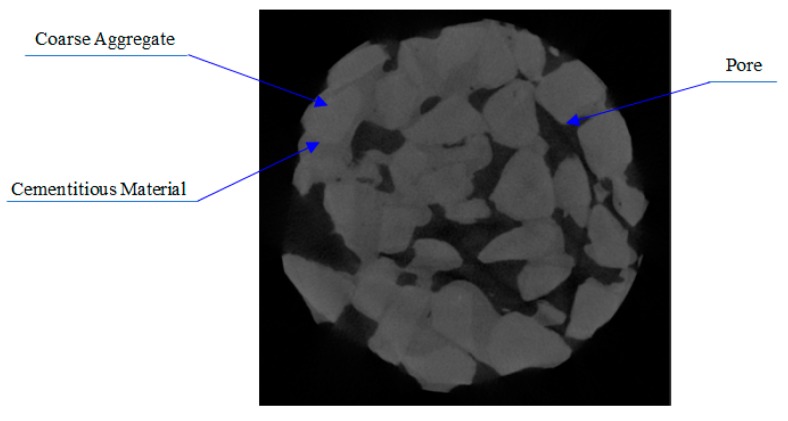
Slice Image of Vegetation Growing Recycled Concrete (VGRC).

From the 3D model images of three groups of specimens, we can clearly see the internal morphological differences of the VGRC with different mix proportions, and the influence of porosity and aggregate size on the internal pore distribution: the larger porosity, the larger pore volume, and the higher the frequency of large pore size; the larger the aggregate size, the more connected the pores, and the sparser the structure. Coarse aggregate, cementitious material, and pores can be distinguished in the model section image (see Figure 22). The recycled aggregate used in VGRC was crushed from waste concrete containing a large amount of cement slurry. CT detection generates different digital information according to the different X-ray absorption coefficients of the different substances, and then generates a volume model. Therefore, the model effect is basically the same for the parts with a similar absorption coefficient, so the distinction between the aggregate and cementitious layers is low.

**Figure 23:**
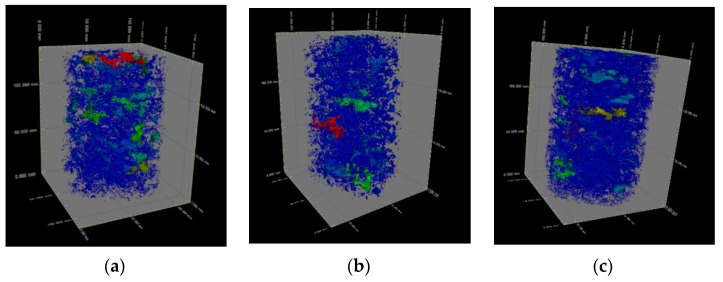
3D Image of Porosity. (**a**) CT-1; (**b**) CT-2; (**c**) CT-3.

**Figure 24:**
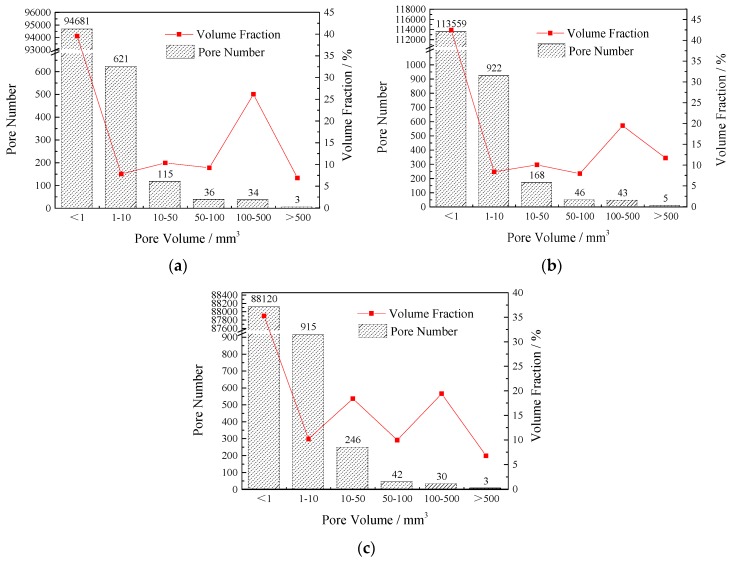
Histogram and Volume Percent of Pore Volume. (**a**) CT-1; (**b**) CT-2; (**c**) CT-3.

A 3D visualization model of the pores can be obtained by 3D reconstruction of VG software (see Figure 23). The parameter information of each pore can be extracted by the defect inspection function. Taking CT-1 as an example, the input results are shown in Table 7. The pore quantity frequency histogram and the percentage of pore volume in each section to total pore volume were obtained by the statistical analysis of the pore volume and pore number of the three samples. The results show (see Figure 24) that the porosity of the concrete is 31.0%, 18.1%, and 28.9%, which is little different from the actual porosity of 30.3%, 19.4%, and 30.4%. The proportion of small pores is basically the same in the different mix ratios. Most of them are capillary pores in the mortar layer and the small pores are identified by edge recognition. The void fraction of 50–500 mm^3^ is increased with the increase of porosity. Continuous grading VGRC has a more uniform pore distribution and a denser structure when compared with large aggregate VGRC.

**Figure 25:**
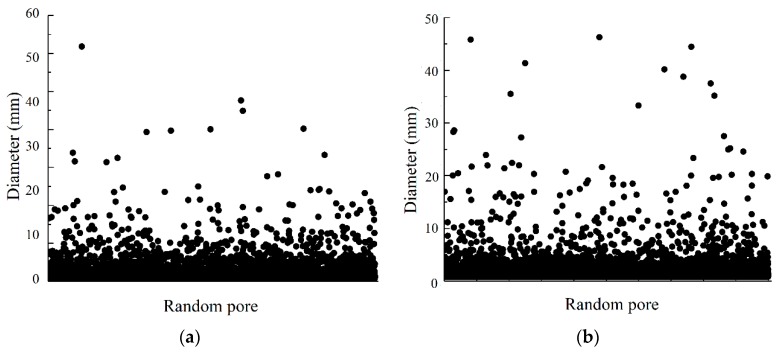

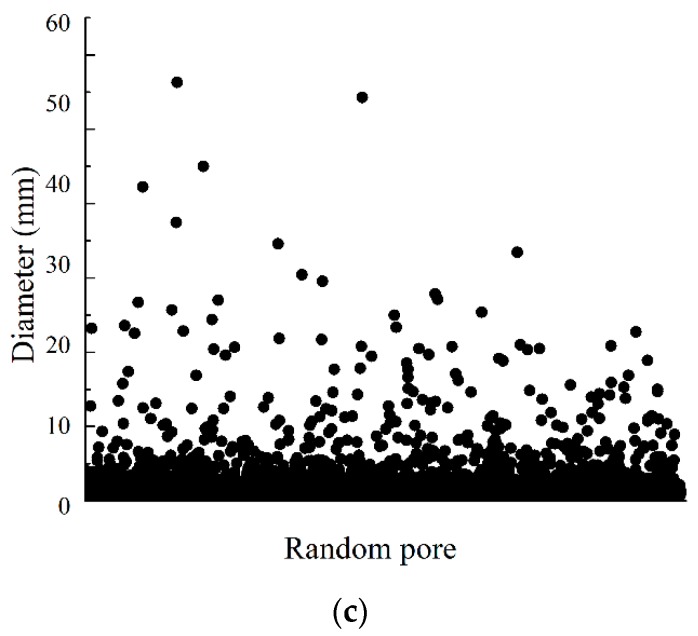
Pore cloth of different diameters. (**a**) CT-1; (**b**) CT-2; (**c**) CT-3.

The pore size distributions of the three specimens are shown in Figure 25. The average pore sizes of the specimens are 0.5653 mm, 0.5781 mm, and 0.5782 mm. There are many micro voids in the vegetation concrete mortar layer, so the effect of the mix ratio on the average pore size is small. Under the conditions of continuous aggregate size gradation and 30% porosity, the pore proportion of 5–10 mm apertures in a specimen is higher than that of the 20% porosity specimen. Under the same porosity conditions, the number of pore sizes larger than 20 mm increases when the gradation is continuous.

### 4.2. Relation between Pore Sphericity and Pore Diameter

The spherical shape is the ideal shape of the pore because of the uniform stress distribution. The closer the VGRC pores are to globularity, the less fracture possibility is in the pore position under loading. Sphericity is the similarity degree of an object’s shape to a sphere. The formula for calculating sphericity is as follows:

(3) φ=vvsa

In the formula, *v* is the object volume, and *v_s_* is the volume of the outer sphere with the object’s longest axis as the diameter.

The relationship between the pore diameter and the sphericity in the three specimens was calculated, and the power function was fitted by the curve fitting toolbox of MATLAB 7.1. When the amount of data is too large, the robust fitting (Robust) method can eliminate outliers; the effect of the outliers is less and the regression coefficient is more robust than other regression methods.

The fitting model is as follows:

(4) $$y=ax^{b}$$

The fitting equation of CT-1 is:

(5) $$y=0.5425x^{-0.3644}$$

The fitting equation of CT-2 is:

(6) $$y=0.5420x^{-0.3616}$$

The fitting equation of CT-3 is:

(7) $$y=0.5423x^{-0.3608}$$

**Figure 26:**
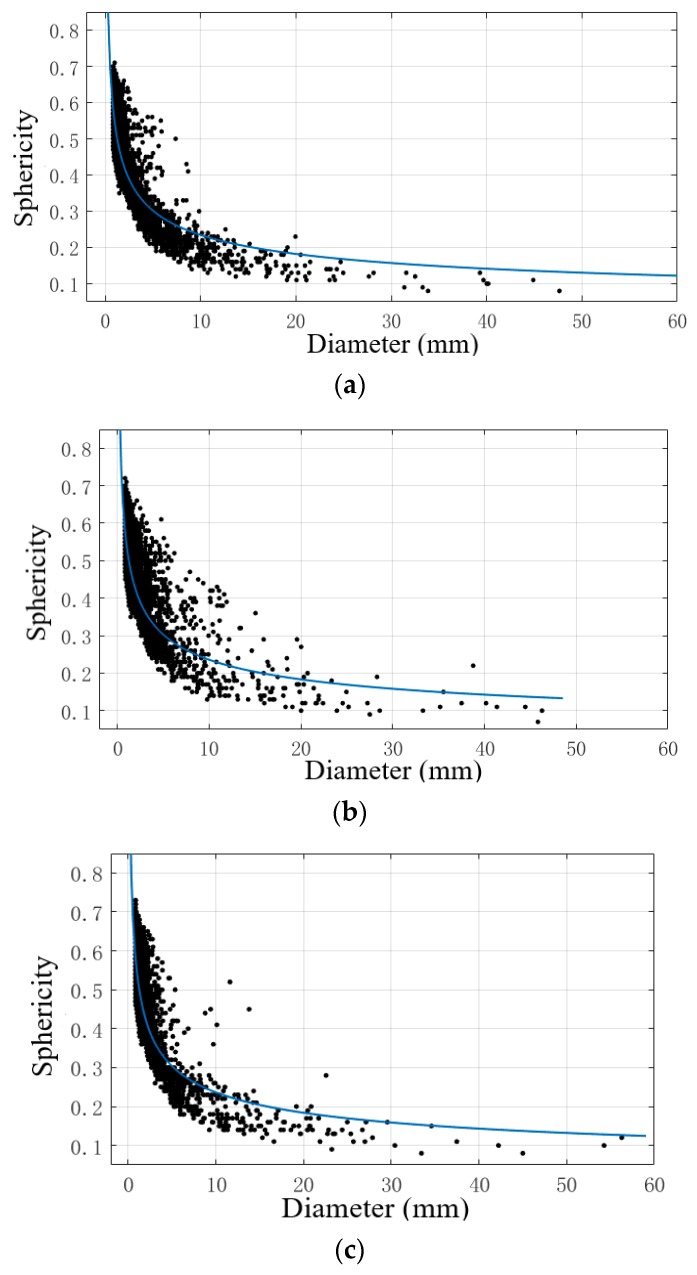
Relationship between pore diameter and sphericity. (**a**) CT-1; (**b**) CT-2; (**c**) CT-3.

The fitting curve is shown in Figure 26. The R-square values are 0.9992, 0.9981, and 0.8949. According to the fitting results, the coefficients *a* and *b* of the three samples are close, indicating that the porosity and aggregate gradation have little influence on the relationship between the pore diameter and sphericity, and they are all power functions. From the relationship between the pore size and sphericity, it can be seen that the pore sphericity decreases with the increase of pore size; thus, the smaller the pore size, the more regular the pore shape, and the maximum sphericity is 0.78. The pore sphericity distribution of the same diameter is larger, and the morphology is unstable when porosity is low.

### 4.3. Pore Plane Distribution Characteristics

**Figure 27:**
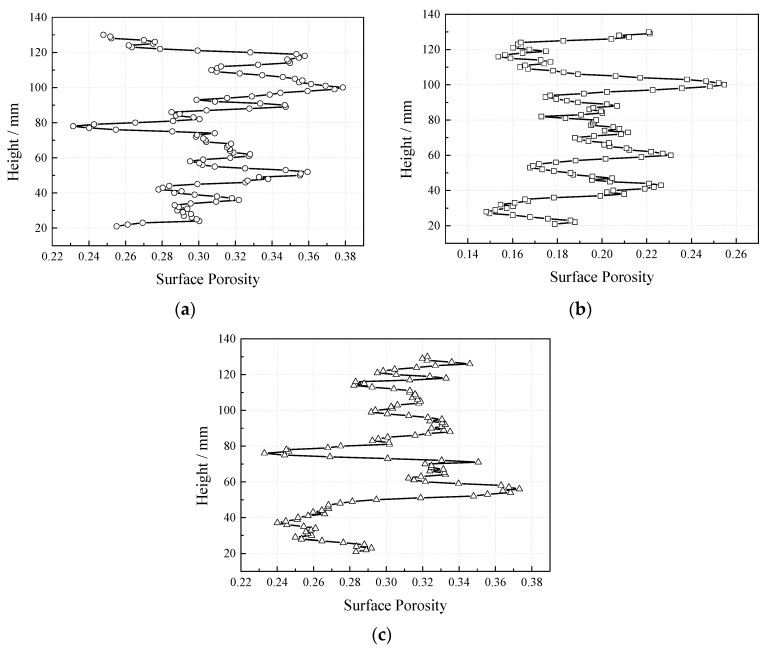
Surface Porosity Distribution of Specimen Height Extension. (**a**) CT-1; (**b**) CT-2; (**c**) CT-3.

Image Pro Plus 6.0 (IPP) is a professional image analysis software developed by the Media Cybernetics Company. It has a powerful image processing and analysis module and abundant customization functions. We used IPP software to process a two-dimensional image of the CT inspection section based on the stereology principle, and analyzed the pore parameters, such as the interface pore area. In the 3D model of the concrete scanned by CT, the uneven part of 20 mm at the upper and lower portion was removed to reduce the result error caused by the deposition of cementitious materials or the unevenness at the top. A total of 110 overlooking sections in BMP format was intercepted with a distance of 1 mm. The measurement parameters of the IPP software were adjusted, the target pore was selected by Automated Optical Inspection (AOI), and batch processing was carried out by macro operation. The broken line graph (see Figure 27) was made of the surface porosity output from IPP and the height. With the increase of porosity, the average surface porosity of each section increases gradually, and the average pore area and the maximum pore area of a single pore in the section also increases. The surface porosity of specimens with different proportions fluctuate around the mean value and has no obvious correlation with the height, which indicates that the properties of the VGRC are uniform and the pore structure is stable.

## 5. Alkali Reduction Treatment of Vegetation Growing Recycled Concrete (VGRC) and Selection of Plant Material

### 5.1. Modification of Alkali Environment in the Pores of Vegetation Growing Recycled Concrete (VGRC)

**Figure 28:**
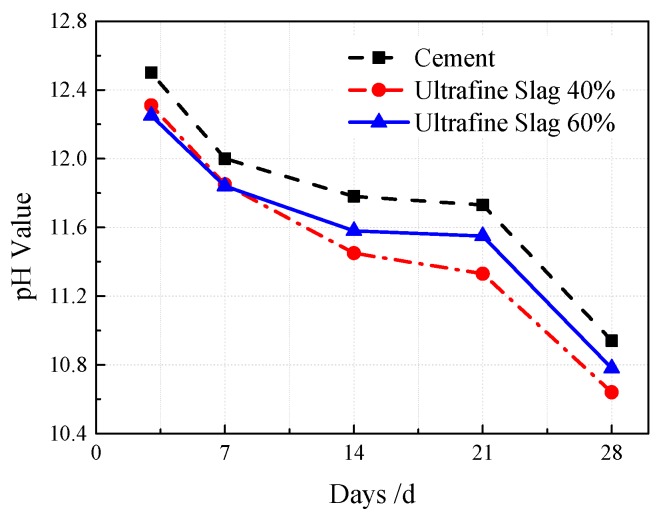
The Influence of Ultrafine Slag Contents on Alkalinity.

Soil pH is generally 3.5–9.5. The existence of Ca(OH)_2_ crystals produced by cement hydration and soluble alkali such as Na and K ions brought in by raw materials for cement production or coal combustion makes the VGRC pore present as alkaline, and the pH value usually ranges from 12 to 13 [27,28], or even more than 14. Therefore, in order to meet the requirements of plant growth, it is necessary to reduce the alkali content of the VGRC. The cement content in cementitious materials was replaced by ultrafine slag in the 40% and 60% proportions. The change of the pH value during the 28 days tested by the alkalinity release method (see Figure 28) shows that the pore alkalinity of concrete with different proportions decreases gradually with the increase of time. The pH value decreases by 0.15–0.40 at 40% ultrafine slag incorporation and 0.16–0.25 at 60% incorporation, indicating that the addition of ultrafine slag can reduce the porosity and alkalinity, but the effect is limited. This is because the main components of ultrafine slag are SiO_2_ and CaO, which can react with the Ca(OH)_2_ produced by cement hydration to produce calcium silicate hydrate with low alkalinity, thus reducing the alkalinity, while the pH value of ultrafine slag is approximately 10, so the alkalinity control effect is not obvious.

**Figure 29:**
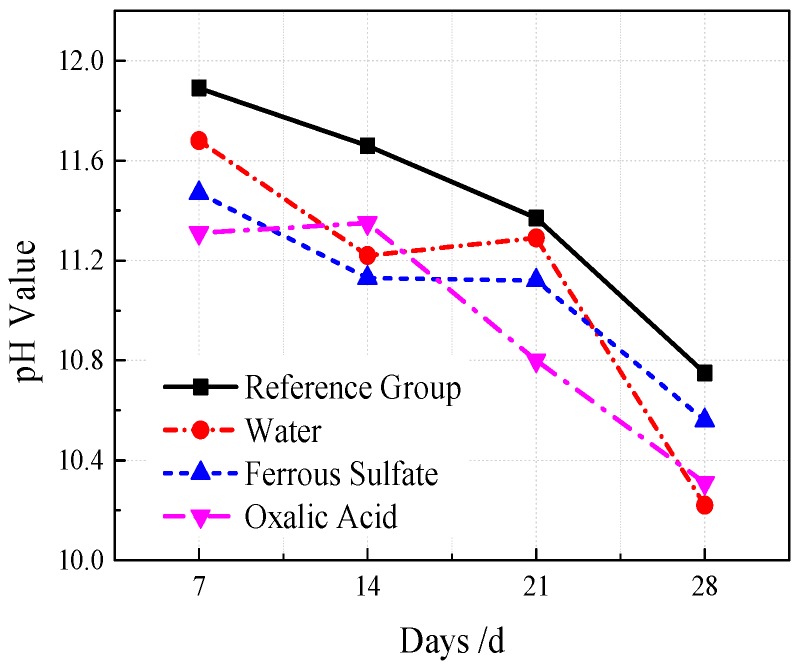
The Effect of Acid Regulator Spraying on pH value.

VGRC was modified with an acid solution based on the neutralization principle of acid and alkali. Clear water, 2 mol/L of an oxalic acid solution, and 1 mol/L of a ferrous sulfate solution were used to spray and soak the VGRC samples, and their effects on the pore pH value and the compressive strength were analyzed. Spraying frequency was every 10 h, and the spraying effect should ensure that the concrete pores be completely wetted. The surface of the test block was wrapped and sealed with plastic film after spraying. The results show (see Figure 29) that the pH value can be reduced by 0.08–0.53 in clear water, 0.25–0.53 in ferrous sulfate, and 0.31–0.57 in oxalic acid. The regulatory effects of the three spraying fluids on pore pH are better than that of natural carbonation, but it is not obvious [29]. The data from the compressive strength test show that the influence of the spraying treatment on the 28-day compressive strength is within the tolerance range. In the spraying treatment, the loss of the spraying fluid is too fast, and the ions reacting with the concrete in the solution are less, so it has little effect on the performance of the cement slurry.

**Figure 30:**
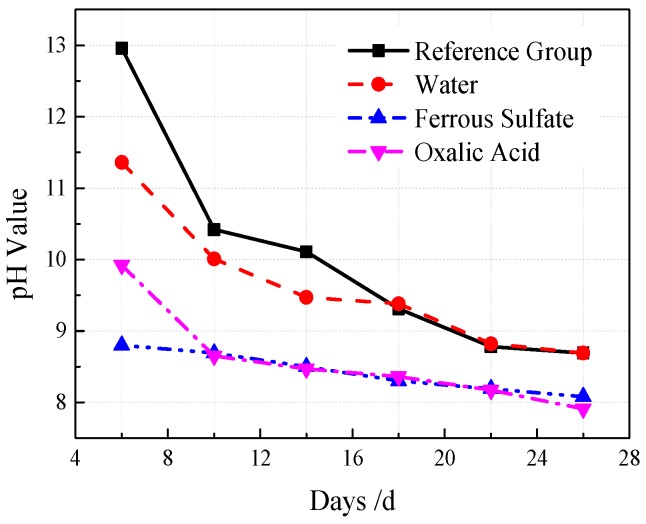
The Effect of Acid Regulator Soaking on pH-value.

During the soaking treatment, a fresh water soaking solution was replaced daily, and the pH value of each group was monitored. According to the compressive strength of the soaked specimens, the strength of the clear water soaking group is 7.5 MPa, 23% higher than that of the reference group; the ferrous sulfate group is 7.1 MPa, 16.4% higher than the reference group; and the oxalic acid group is the same as the reference group. VGRC is a porous structure; the water evaporation is too fast, so water immersion can improve the hydration of the cement. Ferrous sulfate reacts with Ca(OH)_2_ as an acidic solution, which promotes the hydration reaction of the cement to increase Ca(OH)_2_. Therefore, the early strength of cement can be improved slightly [30]. The neutralization of the oxalic acid and alkaline substances reduces the strength of the cement slurry, but the calcium oxalate produced has a certain strength. It can be inferred that when the concentration of oxalic acid is 2 mol/L, the strength loss caused by the corrosion of the cement mortar and the strength increase caused by the formation of calcium oxalate reach a balanced state. The change of the concrete pore alkalinity after immersion with time is made into a broken line chart (see Figure 30). The pore pH value is 8.8 and 8.08 at 6 days and 26 days, respectively, indicating that the solution of ferrous sulfate could react with most alkaline substances within 6 days. The pH value is 9.92 when soaked in oxalic acid for 6 days, which indicates that the alkaline substances are only neutralized but still leached out after a long time soaking; the pH value is 8.65 when soaked for 10 days, and the alkaline substances produced by the cement basically disappeared. The decreasing trend of pH value in the clear water group is similar to that in the reference group, and it decreases to 8.69 in 26 days. Clear water does not react with cementitious components; it can only wash away the alkaline substances precipitated from the surface layer. According to the Lechatelier principle, alkaline ions will move to lower concentration aqueous solutions when the solution is unsaturated. Therefore, the longer the soaking time, the more alkaline substances are dissolved, and the lower the pore alkalinity becomes.

### 5.2. Ratio and Properties of Plant Substrate

**Figure 31:**
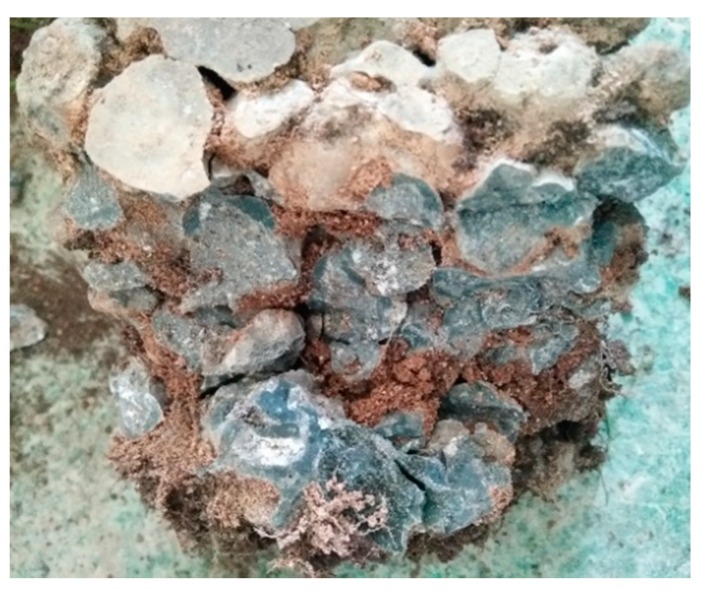
The Filling Effect of Plant Substrate.

The basic skeleton structure of VGRC can provide a growth space for plants, but it lacks the necessary nutrients and moisture for plant growth. Therefore, it is necessary to fill certain growth substrates in the pores to provide a survival carrier for plants. According to the requirements of vegetation for water and fertilizer conservation, balanced ventilation, etc., the experiment divides the plant substrate into two parts: porous base material and surface base material. After mixing the pore substrate (the composition is shown in Table 8) with a nutrient solution (composition is shown in Table 9), the suspended slurry was made and poured into the VGRC inside the connected pores. The surface substrate (composition is shown in Table 10) covered the surface of the VGRC and sowed grass seeds in it. The VGRC was placed in the outdoor site, and the porous substrate was poured into the pores until the substrate flows out from the bottom of concrete; then the base substrate was used to cover the concrete surface. Static for one week, the specimen was taken back after the porous substrate was air-dried, and the specimen was crushed by a press to observe the filling effect of the internal substrate. It can be seen from the cross-section (see Figure 31) that the plant substrate has a good filling effect, and it is basically filled with VGRC pores. The plant substrate is filled into the VGRC specimen without an alkali-reduction treatment and then placed in an outdoor natural environment. The VGRC specimens were crushed to measure the change of the pH values of the plant substrates. The pore pH value of the plant substrate is 10.34, and that of the blank concrete is 11.21. Therefore, the application of a plant substrate can reduce the increase of alkalinity in the pores.

## 6. Compatibility of Vegetation Growing Recycled Concrete (VGRC) and Plants

### 6.1. Lawn Adaptability

**Figure 32:**
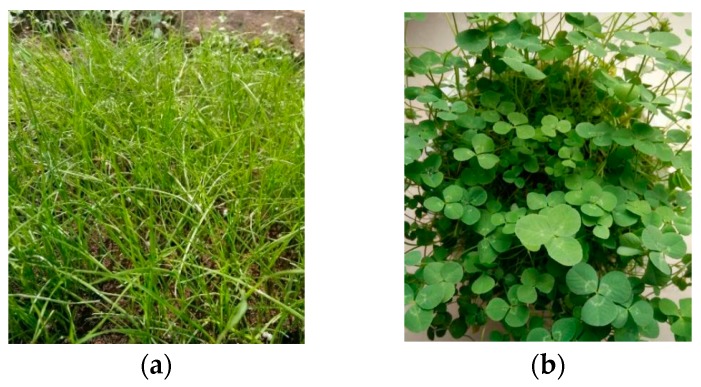

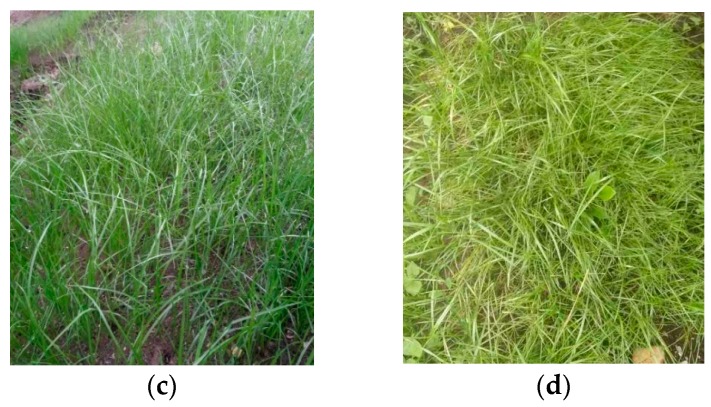
Planting Effect of Different Grass Species. (**a**) Tall fescue; (**b**) White clover; (**c**) Dwarf fescue; (**d**) Ryegrass.

Tall fescue, white clover, ryegrass, and dwarf fescue were planted on the VGRC surface to study the adaptability of different grass species. The planting effect after 30 days is shown in Figure 32. The tall fescue has the fastest turf-forming speed, and the dwarf fescue has better lawn height and landscape. Ryegrass began to wither in a large area from lawn center after being planted for 30 days, and then a large number of grass species died. It can be inferred that ryegrass is not suitable for the dry climate in the north of China or it has a weak alkali resistance.

**Figure 33:**
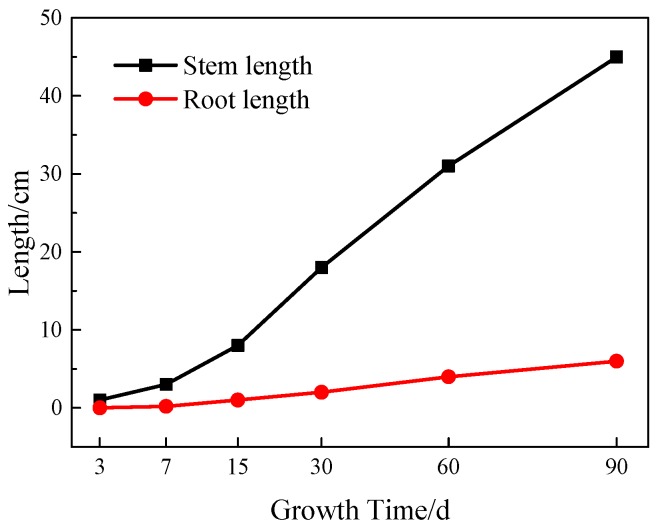
Plant Growth Record.

The leaf height and root growth of tall fescue were measured regularly as an example. According to the data of the growth effect (see Figure 33), tall fescue germinated 3 days after planting, the average height is 8 cm and 15 days after planting; after 30 days, the height is 18 cm and the root length is 4.5 cm, which is basically the same as that of common soil; after 60 days, the height is 31 cm, the average leaf width is 3 mm, the root length is 5.7 cm, and it can be seen that the roots penetrate 6 cm into the VGRC blocks; after 90 days, the height is 45 cm, the leaf width is 4 mm, and the root length is 6.2 cm. Comparing the root morphology of tall fescue in VGRC and common soil: the root system is a taproot growing vertically and downward in common soil, but it is fibrous roots in the VGRC, staggering in the surface and pore substrate. However, the different morphology of two kinds of roots can all satisfy the nutritional supply for the normal growth of plants.

### 6.2. Influencing of Plant Root System on Vegetation Growing Recycled Concrete (VGRC) Performance

**Figure 34:**
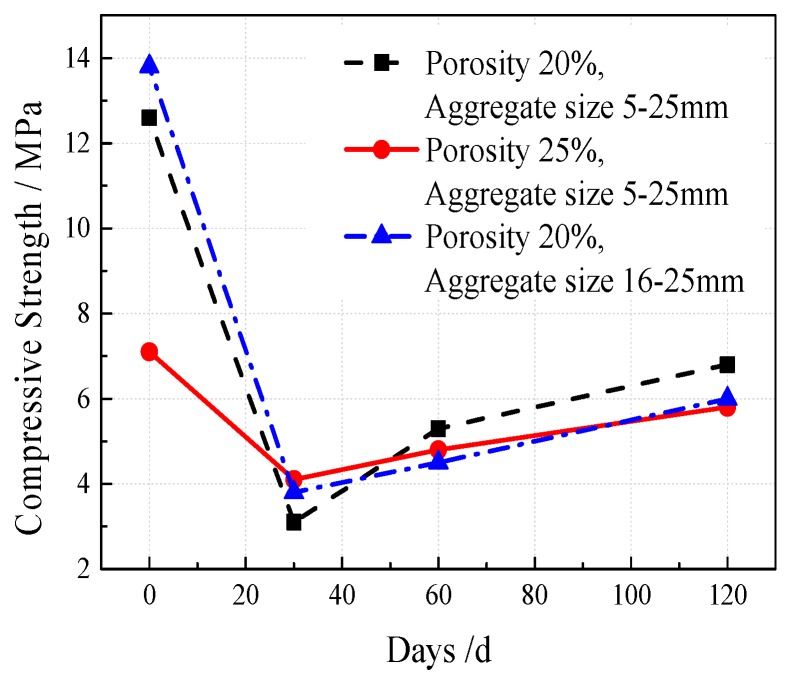
Effects of Plant Growth on Compressive Strength.

The effects of plant roots on the compressive strength of VGRC at different growth stages were analyzed under different mix ratios. The experimental results are shown in Figure 34. The compressive strength decreased by 3–10 MPa after being planted for 30 days, and gradually increased by 1.7–3.7 MPa when planted for 60–120 days. The increase of porosity increases the loss of the compressive strength of the concrete after plant growing, while the aggregate size has little effect on it. Plant substrate easily fills in the effective pores with larger pore size due to the component size and fluidity limitations. Substrate filling is not uniform in low porosity. The filling quantity of the plant substrate is insufficient in some small pores, lacking the protective effect on concrete. In the early planting stage, plants are in the germination and rooting stages, and need frequent irrigation to provide a humid environment. At this time, plants actively absorb nutrients and trace elements in the soil. A certain amount of acid secretion is released and accompanied by flowing water intruding into the cement paste layer. It leads to the alkali–aggregate reaction of the recycled aggregates, which affects the compressive strength. With the increase of growth time, the water requirement and root exudate effect decreases gradually. The compressive strength of concrete is a continuous growth process. If the period of the root system is not strong enough, and it can not fill the VGRC pores because the root diameter is smaller than the pore diameter and the root system will not exert mechanical pressure on the concrete. Therefore, the strength has rebounded.

**Figure 35:**
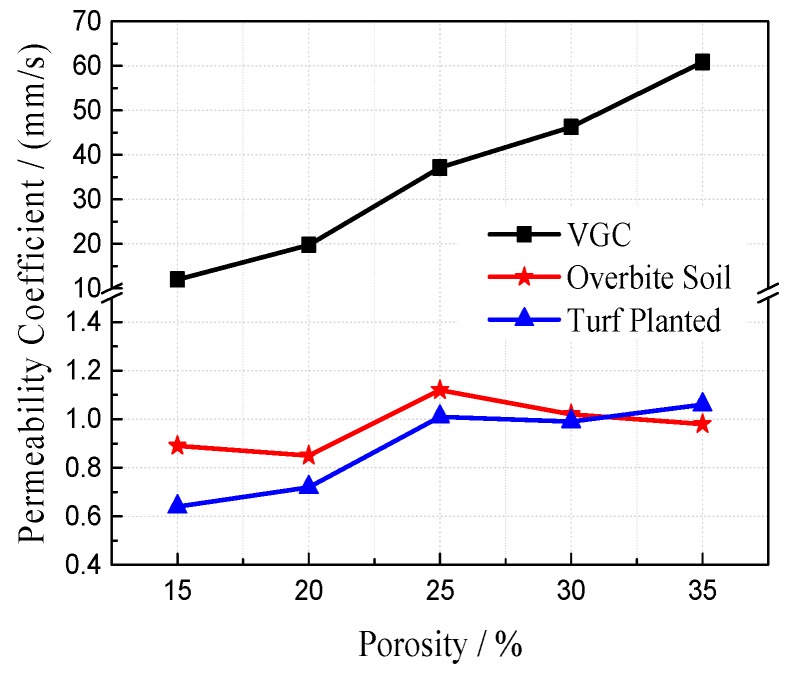
Effects of Plant on Water Permeability in Different Porosity.

**Figure 36:**
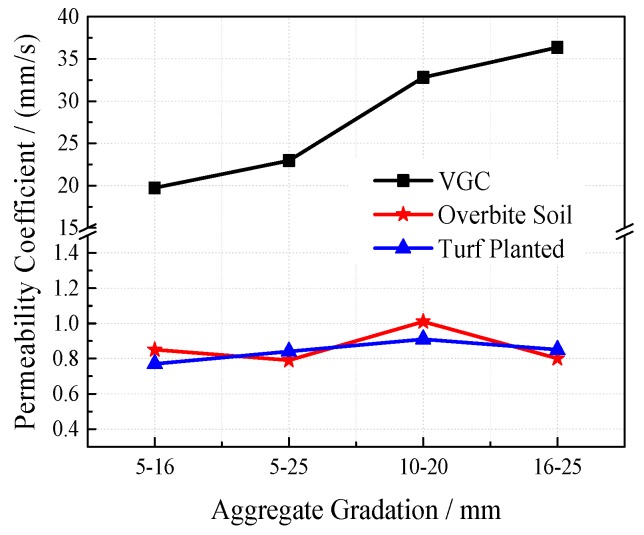
Effects of Plant on Water Permeability in Different Aggregate Gradation.

The permeability coefficient is a direct and effective index to characterize the permeability of porous concrete. To study the effect of the planting process on the permeability of VGRC, experiments were set up under different porosity and aggregate gradation. The permeability coefficients of the VGRC, using a plant substrate block (i.e., overbite soil), and when the specimens have been planted for 30 days (i.e., turf planted) increase with the increase of porosity (see Figure 35 and Figure 36). Therefore, design porosity is the decisive factor affecting the permeability of VGRC. Water permeability after turf has been planted is basically the same as that after overbite soil. The permeability coefficient decreases greatly but is still higher than common soil. Thus, the VGRC can not only realize the ecological function of drainage and permeability but can also ensure the growth of plants.

**Figure 37:**
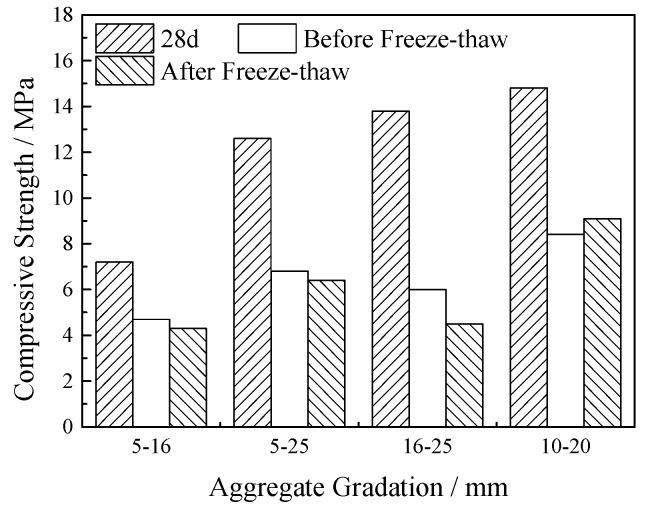
Effect of Aggregate Gradation on Frost Resistance after Planting.

**Figure 38:**
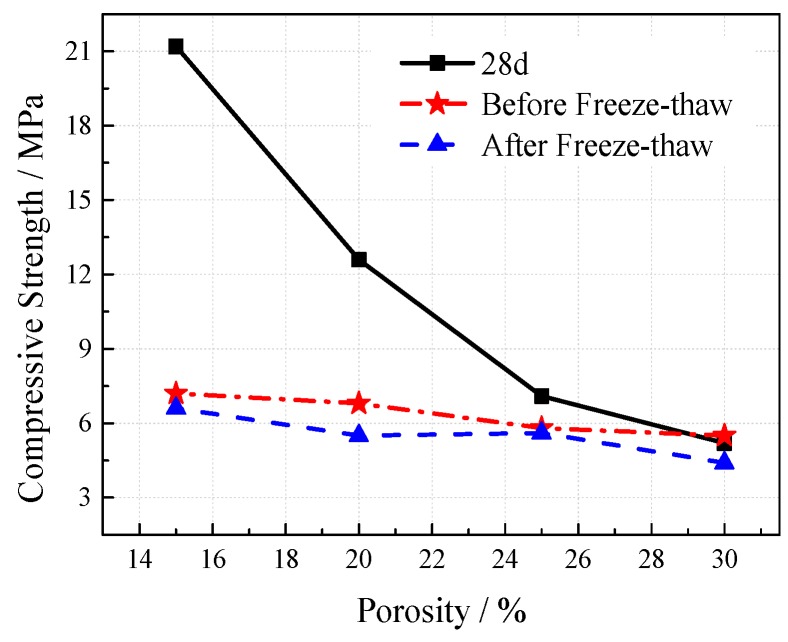
Effect of Porosity on Frost Resistance after Planting.

The frost resistance test of the VGRC was carried out with different aggregate gradation and porosity. The test results are shown in Figure 37 and Figure 38. After 120 days of turf planting, the compressive strength of VGRC with different aggregate sizes decreased significantly when compared with the initial strength. The weakening degree of concrete strength by the plants decreases with the increase of porosity. After freeze-thaw cycles, the compressive strength of the concrete blocks planted with lawn did not decrease significantly when compared with that before freeze-thaw cycles. It can be seen that the synergistic effect of vegetation and concrete can enhance the frost resistance of VGRC, so that the freeze-thaw damage is no longer the main factor affecting the compressive strength.

## 7. Conclusions

The study was carried out in Shenyang, Liaoning Province, China, where the climate is temperate and semi-humid continental and the vegetation types in this study are mainly suitable for this climate. The following conclusions could be drawn:

(1) The strength of cement mortar increases first and then decreases with the increase of ultrafine slag replacement rate. The XRD analysis shows that the content of Ca(OH)_2_ in the hydration products of 28-days cement mortar decreases significantly with the addition of ultrafine slag. The unhydrated ultrafine slag has a filling micropore function.
(2) Porosity plays a decisive role among many factors affecting the compressive strength of VGRC. The compressive strength decreases gradually with the increase of porosity. The compressive strength and planting demand can be satisfied when the porosity is 20%–25%. The aggregate quality and cementitious strength are the main factors affecting compressive strength under the large porosity, while the influence of the aggregate size and water–cement ratio is small. The strength of cementitious materials can be improved by adding ultrafine slag, and the optimum dosage is 40%.
(3) The compressive strength of VGRC is greatly damaged by the fast freezing method. After 50 freeze-thaw cycles, the strength loss rate is 8%–55%. However, the degree of damage of natural freeze-thaw is relatively small.
(4) The alkaline environment in the concrete pores is mainly caused by alkaline substances in the cement mortar. The pore pH value can be reduced by 0.16–0.57 by changing the composition of the cementitious materials and spraying a modified solution. The pore pH value can be reduced to below 8 by ferrous sulfate solution soaking of 6 days, oxalic acid solution soaking of 10 days, or water soaking of 26 days.
(5) Most grass species such as tall fescue can grow normally in vegetation concrete. The roots can penetrate 6-cm thick VGRC blocks after being planted for 60 days.
(6) The compressive strength of the VGRC decreases greatly after turf planting, and the increase in porosity can reduce the weakening effect of vegetation on the strength. The permeability coefficient of VGRC increases with the increase of porosity and aggregate size. The permeability coefficient decreases greatly after turf planting and overbite soil but is still higher than that of common soil. After being planted for 120 days, the effect of freeze-thaw cycles on the concrete strength decreases, and the aggregate size and porosity have no significant correlation with the frost resistance.

## Figures and Tables

**Table 1:** Aggregate Basic Performance.

Aggregate Type	Aggregate Size/mm	Apparent Density/(kg/m^3^)	Bulk Density/(kg/m^3^)	Crushing Index/%	Water Absorption of 10 min/%
Recycled Aggregate	5–25	2390	1332	17.9	4.76
Ordinary Aggregate	5–25	2520	1470	9.3	1.92

**Table 2:** Chemical Composition of Ultra-fine Slag/%.

Composition	Na_2_O	MgO	Al_2_O_3_	SiO_2_	K_2_O	CaO	Fe_2_O_3_	SO_3_	Loss of Ignition
Content	0.3	4.38	13.58	30.78	0.82	44.61	0.92	3.08	1.08

**Table 3:** Mixture Proportions of Specimens.

Group	Water Cement Ratio	Porosity (%)	Cement (kg/m^3^)	Slag (kg/m^3^)	Water (kg/m^3^)	Coarse Aggregate (kg/m^3^)	Water Reducer (g/m^3^)
A1	0.23	20	185.3	278	106.6	1305	3.24
A2	0.25	20	179	268.5	111.9	1305	2.46
A3	0.27	20	173	259.6	116.8	1305	2.16
A4	0.29	20	167.5	251.2	121.4	1305	1.88
B1	0.25	15	160.5	240.8	100.3	1305	2.2
B2	0.25	20	134.8	202	84.3	1305	1.85
B3	0.25	25	109	153.5	68.3	1305	1.63
B4	0.25	30	83.3	125	52	1305	1.18
C1	0.25	25	508.8	0	127.2	1323	2.29
C2	0.25	25	407.0	101.8	127.2	1323	1.83
C3	0.25	25	305.3	203.5	127.2	1323	1.37
C4	0.25	25	203.5	305.3	127.2	1323	0.92
C5	0.25	25	101.8	407	127.2	1323	0.46

**Table 4:** Ratio and Loss Late of Mass and Strength in Freeze–thaw Test.

Group	Water Cement Ratio	Porosity/%	28d Compressive Strength/MPa	Mass Loss Rate/%		Strength Loss Rate/%	
				25 Times	50 Times	25 Times	50 Times
A1	0.23	20	8.0	1.69	3.92	33.85	55.20
A2	0.25	20	12.9	2.46	2.74	35.21	38.05
A3	0.27	20	15.6	1.24	1.92	39.15	46.51
A4	0.29	20	13.2	0.98	5.96	43.36	54.61
B1	0.25	15	24.5	0.01	1.03	26.65	39.67
B2	0.25	20	12.9	2.46	2.74	35.21	38.05
B3	0.25	25	7.1	2.75	0.64	2.82	8.31
B4	0.25	30	5.8	4.34	11.90	8.65	14.42

**Table 5:** The Mix Proportion of Aperture Analysis Specimen.

Group	Aggregate Size/mm	Porosity/%	Water Cement Ratio	Ultrafine Incorporation
CT-1	16~25	30	0.25	40%
CT-2	5~25	20	0.25	40%
CT-3	5~25	30	0.25	40%

**Table 6:** Equipment Parameters of X-ray Tomography Coordinate Measuring Machine.

Manufacturer	Model	Ray Type	Ray Source	Energy/kv	Max Imaging Pixel	Contrast Ratio/%	Resolution/%	Max Spatial Resolution/mm	Typical Detection Time/min
Germany Werth Company	TomoScope HV Compact225	X ray	Cermet X-ray tube	225	1024*1024	<1	<1	0.02	20

**Table 7:** Measurement Result of CT-1 Porosity.

Number	Diameter/mm	Volume/mm^3^	Voxel/ind	Surface Area/mm^2^	Location x/pixel	Location y/pixel	Location z/pixel
1	44.996	731.73	180503	4798.069	314	358	822
2	22.562	559.08	137914	1178.403	270	265	856
3	54.257	539.55	133095	3172.000	404	548	180
4	33.431	469.41	115795	3527.377	430	191	512
5	30.408	456.99	112731	2937.845	313	579	749
6	27.851	381.86	94198	2199.176	167	180	578
7	56.281	367.49	90653	2114.819	455	492	418
8	42.221	288.06	71059	2104.701	530	387	588
9	34.574	257.56	63536	1320.116	515	537	348
10	19.621	254.63	62812	1499.864	330	235	579
				……			
94111	1.372	0.21	53	3.356	468	515	524

**Table 8:** The Composition and Mass Fraction of Pore Substrate.

Coir Dust	Fly Ash	Organic Fertilizer	Peat Soil	Cement	Water Retaining Agent
45%~58%	17%~23%	20%~25%	5%~10%	5%~7%	0.2%~0.5%

**Table 9:** The Composition and Mass Fraction of Surface Substrate.

Peat Soil	Coir Dust	Organic Fertilizer	Agricultural Water Retaining Agent
65%~80%	20%~30%	3%~5%	2%~3%

**Table 10:** The Composition and Mass Fraction of Nutrient Solution.

Sucrose	Aspirin (Acetylsalicylic Acid)	Auxin ^1^		Potassium Dihydrogen Phosphate
5~10g	0.4~0.5g	Iaa 0.3g	Naa 0.5g	27g

^1^ Auxin should be dissolved in 95% ethanol solution.

## References

[1] Sumanasooriya M.S., Neithalath N. (2011). Pore structure features of pervious concretes proportioned for desired porosities and their performance prediction. Cem. Concr. Comp..

[2] Okamoto T., Masui N. (2000). Manufacture of porous concrete. Korea Concr. Inst..

[3] Bhutta M.A.R., Tsuruta K., Mirza J. (2012). Evaluation of high-performance porous concrete properties. Constr. Build. Mater..

[4] Kevern J.T., Nowasell Q.C. (2018). Internal curing of pervious concrete using lightweight aggregates. Constr. Build. Mater..

[5] Kyung H.L., Keun H.Y. (2016). Development of a neutral cementitious material to promote vegetation concrete. Constr. Build. Mater..

[6] Mohammed B.S., Liew M.S., Alaloul W.S. (2018). Properties of Nano-Silica Modified Pervious Concrete. Case Stud. Constr. Mater..

[7] Oh R.O., Cha S.S., Park C.G. (2014). Mechanical properties and water purification characteristics of natural jute fiber-reinforced non-cement alkali-activated porous vegetation blocks. Paddy Water Environ..

[8] Ghashghaei H.T., Hassani A. (2016). Investigating the relationship between porosity and permeability coefficient for pervious concrete pavement by statistical modeling. Mater. Sci. Appl..

[9] Huang B., Wu H., Shu X. (2010). Laboratory evaluation of permeability and strength of polymer-modified pervious concrete. Constr. Build. Mater..

[10] Lian C., Zhuge Y., Beecham S. (2011). The relationship between porosity and strength for porous concrete. Constr. Build. Mater..

[11] Jang J.G., Ahn Y.B., Souri H., Lee H.K. (2015). A novel eco-friendly porous concrete fabricated with coal ash and geopolymeric binder: Heavy metal leaching characteristics and compressive strength. Constr. Build. Mater..

[12] Deo O., Neithalath N. (2011). Compressive response of pervious concretes proportioned for desired porosities. Constr. Build. Mater..

[13] Codina M., Cau-dit-Coumes C., Bescop P.L., Verdier J., Ollivier J.P. (2007). Design and characterization of low-heat and low-alkalinity cements. Cem. Concr. Res..

[14] Qi Q. (2018). Experimental Study and Application of Forst-Resistant Vegetation Concrete Alkaline-Reducing Planting Technology. Master’s Thesis.

[15] Räsänen V., Penttala V. (2003). The pH measurement of concrete and smoothing mortar using a concrete powder suspension. Cem. Concr. Res..

[16] Wong N.H., Chen Y., Ong C.L., Sia A. (2003). Investigation of thermal benefits of rooftop garden in the tropical environment. Build Environ..

[17] Kim Y., Hanif A., Kazmi S.M.S. (2018). Properties enhancement of recycled aggregate concrete through pretreatment of coarse aggregates–Comparative assessment of assorted techniques. J. Clean. Prod..

[18] Zhao Z., Remond S., Damidot D. (2015). Influence of fine recycled concrete aggregates on the properties of mortars. Constr. Build. Mater..

[19] Guo H., Shi C., Guan X. (2018). Durability of recycled aggregate concrete–a review. Cem. Conc. Compos..

[20] Yap S.P., Chen P.Z.C., Goh Y. (2018). Characterization of pervious concrete with blended natural aggregate and recycled concrete aggregates. J. Clean. Prod..

[21] Rahman M.A., Imteaz M.A., Arulrajah A. (2015). Recycled construction and demolition materials in permeable pavement systems: Geotechnical and hydraulic characteristics. J. Clean. Prod..

[22] Gelong X., Weiguo S., Bingliu Z. (2018). Properties of recycled aggregate concrete prepared with scattering-filling coarse aggregate process. Cem. Conc. Compos..

[23] Yang J., Jiang G. (2003). Experimental study on properties of pervious concrete pavement materials. Cem. Concr. Res..

[24] Yan X., Gong C., Wang S. (2013). Effect of aggregate coating thickness on pore structure features and properties of porous ecological concrete. Mag. Concr. Res..

[25] Ji B.S. (2013). Experimental Study on Microporous Ecological Concrete. Master’s Thesis.

[26] Taniguchi S., Nishizaki I., Moriyoshi A. (2008). A study of longitudinal cracking in asphalt pavement using CT scanner. Road Mater. Pavement..

[27] Bameyback R.S., Diamond S. (1981). Expression and analysis of pore fluids from hardened cement pastes and mortars. Cem. Concr. Res..

[28] Taylor H.F.W. (1987). A method for predicting alkali ion concrete reactions in cement pore solutions. Adv. Cem. Res..

[29] Ahn T.W., Choi I.S., Oh J.M. (2008). A study on water quality purification function by using planting concrete and porous concrete. Environ. Impact Assess..

[30] Zhao J. (2017). Research on Reduction of Alkali and Protective Slope of the Recycled Brick Aggregate Porous Concrete. Master’s Thesis.

